# Examining Public Sector Availability and Supply Chain Management Practices for Malaria Commodities: Findings From Northern Nigeria

**DOI:** 10.9745/GHSP-D-22-00547

**Published:** 2024-06-27

**Authors:** Kunle Rotimi, Ademola Joshua Itiola, Babatunde Ariyo Fagbemi, Jimmy Aiden, Taiwo Ibinaiye, Chrysantus Dabes, Andrew Okwulu, Daniel Oguche, Adaeze Aidenagbon, Umar Babuga Abubakar, Rose Tukura, Danladi Chonoko Mohammad, Christopher Bewa, Ahmad Muhammad Danzaki, Olusola Oresanya

**Affiliations:** a Malaria Consortium, Abuja, Nigeria.; b School of Public Health, University of Alberta, Edmonton, Canada.; c Bauchi State Agency for the Control of HIV/AIDS, Tuberculosis/Leprosy and Malaria, Bauchi, Nigeria.; d State Logistics Management Coordinating Unit, Kogi State Ministry of Health, Lokoja, Nigeria.; e State Logistics Management Coordinating Units, Kebbi State Ministry of Health, Birnin-Kebbi, Nigeria.; f State Malaria Elimination Programme, Plateau State Ministry of Health, Jos, Nigeria.; g State Logistics Management Coordinating Units, Sokoto State Ministry of Health, Sokoto, Nigeria.

## Abstract

**Background:**

Nigeria accounts for substantial proportions of global malaria infections and deaths, with children aged younger than 5 years being the most affected group. This suggests that access to lifesaving malaria interventions could be suboptimal, especially at public health facilities where most rural dwellers seek health care. We conducted this study to ascertain if public health facilities have the commodities and the robust supply chain management (SCM) system required to deliver malaria interventions to children younger than 5 years.

**Method:**

We conducted a cross-sectional survey in 1,858 health facilities across 7 states in Nigeria. Using structured questionnaires, we assessed the availability of selected malaria commodities required by children aged younger than 5 years. We also interviewed health workers to evaluate other core SCM activities.

**Result:**

More than 50% of health facilities in 5 states were stocked out of malaria rapid diagnostic tests (mRDTs), and stock-out rates for artemisinin-based combination therapies (ACTs) were over 50% for almost all assessed ACTs across all states. The percentage of health facilities that received malaria commodities within the recommended lead time was below average across most states (71%). States with a higher percentage of health workers who were aware of and placed orders following the national reporting timeline and those that delivered commodities to the last mile predominantly through third-party logistics service providers tended to have higher availability of mRDTs and artemether/lumefantrine combinations. The top 2 logistics challenges were insecurity and inadequate funding.

**Conclusion:**

The availability of lifesaving malaria commodities across the health facilities visited was suboptimal, possibly due to several SCM challenges. The results from this study underscore the urgent need to implement effective interventions to address the observed gaps. This will contribute to reducing malaria morbidity and mortality among children aged younger than 5 years in Nigeria.

## Abstract (precis)

The supply management challenges identified in this study underscore the urgent need to implement effective interventions to address the observed gaps in malaria commodity availability to help reduce malaria morbidity and mortality in Nigeria, especially among children aged younger than 5 years.

## Teaser

**Key Findings:**

- The persistence of malaria as a leading cause of mortality and morbidity in Nigeria, especially in children aged younger than 5 years, may be related to factors that result in a weak public sector supply chain management (SCM) system.
- An assessment of malaria commodities availability in 7 states in northern Nigeria found that more than 50% of health facilities in 5 states had stock-outs of malaria rapid diagnostic tests and, in all states, stock-out rates of most artemisinin-based combination therapies were over 50%.
- The poor availability of these commodities highlights the need to strengthen coordination and supply chain workforce capacity and organization to ensure that SCM best practices and standard procedures are followed consistently.

**Key Implications:**

- Local and international stakeholders need to invest more in strengthening the public health supply chain, especially at the subnational level, to reduce malaria-related morbidity and mortality among children aged younger than 5 years in Nigeria.
- The logistics management coordination units should be adequately funded to enhance their effectiveness and improve commodity management.
- Using 3PL service providers for distribution could improve commodity availability and should be encouraged where it is deemed sustainable, given the funding and oversight capacity requirements.

## BACKGROUND

To ensure uninterrupted access to lifesaving commodities, several donors have invested significantly in public health supply chain management (SCM) interventions across low- and middle-income countries.^1^^–^^3^ Despite these numerous investments, commodity availability— especially at the point of service delivery—remains suboptimal, suggesting the need for further supply chain system strengthening interventions.^4^

Nigeria is a major beneficiary of donor support, and several local and international nongovernmental organizations work with the government to implement SCM-related interventions across public health programs, including malaria, HIV/AIDS, and TB. Although the country has recorded some successes with this support, Nigeria still lags in achieving health-related development goals. For example, between 1990 and 2021,^5^ the under-5 mortality rate decreased by nearly half. However, in 2021, the under-5 mortality rate of 110.8 per 1,000 live births remains one of the highest in the world, suggesting that not accelerating the current rate of progress can put the achievement of the Sustainable Development Goal target at risk.^6^^,^^7^

Malaria is one of the leading causes of mortality and morbidity among children aged younger than 5 years in Nigeria, accounting for nearly 36% of deaths of children aged younger than 5 years.^8^^–^^10^ The persistence of malaria as a public health threat may be linked to the weakness of the malaria SCM system, resulting in poor access to health commodities. Several studies and assessments have shown that factors such as poor coordination, human resources capacity gap, inadequate financial resources, and poor-quality data contribute to poor access to medicines, including malaria products, in Nigeria.^11^^–^^13^

The persistence of malaria as a public health threat in Nigeria may be linked to the weakness of the malaria SCM system, resulting in poor access to health commodities.

Developing and implementing evidence-informed malaria interventions require a holistic understanding of the status of SCM of malaria commodities. So far, studies that assessed public health commodity management in Nigeria focused on a few states and did not cover the full breadth of the SCM functions.^14^^–^^17^ Additionally, there is no recent study on the availability of under-5 malaria commodities within the Nigeria public health system.

One main challenge with malaria commodity management in Nigeria’s public sector is the overreliance on donor funding, putting the sustainability of services at risk (Box).^18^^–^^22^ Donors are mostly responsible for commodity procurement, payment of private sector managers of zonal warehouses, distribution of commodities to the last mile, and maintenance of the electronic LMIS platform.

BOX Overview of the Public Sector Malaria Commodity Supply Chain System in Nigeria

The National Malaria Elimination Programme coordinates the activities of all partners and stakeholders involved in malaria control activities in Nigeria, including the facilitation of policy and guidelines development.^18^ Donors, such as the U.S. President’s Malaria Initiative and The Global Fund, support the government’s effort through implementing partners and international development partners, including Chemonics International, the Malaria Consortium, Catholic Relief Services, and Management Science for Health.^19^^–^^21^

All malaria-related supply chain activities are coordinated by the National Malaria Elimination Programme procurement and supply management technical working group (PSM TWG) that collaborates with the integrated PSM TWG for all public health programs in Nigeria. At the state level, the integrated PSM TWG coordinates all supply chain management (SCM) activities for public health programs, including malaria. The operational SCM activities in the country, including logistics management information system (LMIS) reports collection, review, analysis, and aggregation, are coordinated through logistics management coordinating units at the national, state, and local government levels.^22^

Currently, most donor-funded malaria commodities are directly distributed to health facilities from the 6 central warehouses, 2 of which (Abuja and Lagos) serve as the main hub of primary receipt and central storage. Distribution takes place from these warehouses located across the 6 geographical zones in Nigeria using third-party logistics (3PL) service providers, which are private logistic companies offering outsourced logistics services such as transportation. For commodities procured by the state government or donated by other partners, the last mile distribution models vary across the country, from outsourced delivery using 3PL service providers to commodity pickup by health facility staff from the state or local government area store.

Nigeria uses a forced ordering min-max (pull system) inventory control system with maximum and minimum stock levels of 4 months and 2 months, respectively, at the health facilities. To facilitate resupply, health facility staff place orders bimonthly using a bimonthly facility stock status report to bring their stock level to the recommended maximum of 4 months. In line with this inventory policy, the stock level at the health facilities is not expected to fall below the minimum of 2 months when placing an order. However, when the stock level drops to 2 weeks before the end of the review period, health facilities can place an emergency order. The country now uses an electronic LMIS, known as the national health logistics management information system, to collect, aggregate, and analyze LMIS reports across all public health programs, including malaria.^22^

Weakness in human resource capacity, especially at health facilities, also persists, partly because most technical support is concentrated at national and state levels. This is compounded by poor personnel remuneration and a lack of up-to-date knowledge and training.^23^ Other factors, such as poor coordination, poor data quality and availability, and lack of a financial management system, prevent the SCM from functioning optimally.

To provide the required evidence for system strengthening, we assessed the availability of malaria commodities across 7 northern states in Nigeria. We also evaluated other aspects of the SCM needed for the uninterrupted delivery of health commodities.

## METHODS

### Study Area and Period

This study was conducted in 7 northern Nigeria states that were selected with consideration for geopolitical representativeness (i.e., coverage of the 3 northern geopolitical zones). Kogi, Nasarawa, and Plateau states were selected from the north-central region, Kebbi and Sokoto states from the northwest, and Bauchi and Borno states were selected from the northeast geopolitical zone.

### Study Design

This is a cross-sectional descriptive study using both quantitative and qualitative research methods. The recommended method for conducting an assessment with the logistics indicator assessment tool and logistics systems assessment tool^24^^,^^25^ was followed with some adaptations to both the method and the tools (Supplement). All assessment questions followed the phrasing captured in both of these tools.

The National Malaria Elimination Programme in Nigeria recommends a test and treat strategy. This involves testing all suspected malaria cases or fever episodes using malaria rapid diagnostics tests (mRDTs) before treatment with artemisinin-based combination therapies (ACTs).^26^^–^^28^ Artemether/lumefantrine (AL) is the recommended first-line treatment for uncomplicated malaria, with artesunate/amodiaquine (A/A) and dihydroartemisinin/piperaquine (DHP) as alternatives.^27^ Other available ACTs in Nigeria for the treatment of uncomplicated include artesunate/pyronaridine, artesunate/mefloquine, and artemisinin/piperaquine.^26^ We assessed the availability of: artemether 20 mg + lumefantrine 120 mg, 1 × 6 tablets (AL1); artemether 20 mg + lumefantrine 120 mg, 1 × 12 tablets (AL2); artesunate 25 mg + amodiaquine 67.5 mg, 1 × 3 Tablets (AA1); artesunate 50 mg + amodiaquine 135 mg, 1 × 3 Tablets (AA2); and mRDTs.

### Sample Size

By applying the World Health Organization’s recommendations for conducting service availability and readiness assessment,^29^ with a confidence interval of 95% and 5% margin of error, we estimated a sample size of 1,858 health facilities.

### Sampling Procedure

All the state logistics management coordinative units (LMCUs) were sampled for this study. Similarly, all the local government areas (LGAs) across all states were selected, except in Kogi, where some LGAs were excluded due to the absence of malaria public health programs involving mass drug distribution like the seasonal malaria chemoprevention campaigns, which only focus on LGAs with seasonal malaria transmission.

To select health facilities for the study, first, we obtained the list of primary health care centers participating in the malaria public health program across all sampled LGAs from the state malaria elimination program and the state primary health care development agency. Health facilities were then ranked according to the volume of stock they received. Those with the highest allocation were purposively selected until the desired sample size for each LGA was reached. The final sample size across the 7 states is presented in Table 1.

**TABLE 1.** Distribution of Assessed Facilities Across Seven States in Nigeria

	**State LMCU**	**LGA LMCUs**	**Public Health Facilities**
Borno	1	20	200
Bauchi	1	23	337
Kogi	1	9	182
Kebbi	1	21	296
Nasarawa	1	13	252
Sokoto	1	23	299
Plateau	1	23	292

Abbreviations: LGA, local government area; LMCU, logistics management coordinating unit.

### Data Collection Instruments and Procedure

A total of 1,997 respondents were interviewed for the study. Before the commencement of data collection, stakeholders at the state and LGA primary health care development agencies were engaged to obtain administrative approval. Following this approval, trained data collectors administered the electronic copy (using the SurveyCTO application) of the data collection instruments to interview LMCU coordinators at the state and LGA levels and heads of health facilities. Each data collector was assigned to only 1 LGA. The data collection process was closely supervised by a state-level supervisor (1 per state) who also conducted the state-level assessment. All data collection was carried out in January 2022. Participants gave their consent to participate in the study.

### Conceptual Framework and Outcomes

**FIGURE 1:**
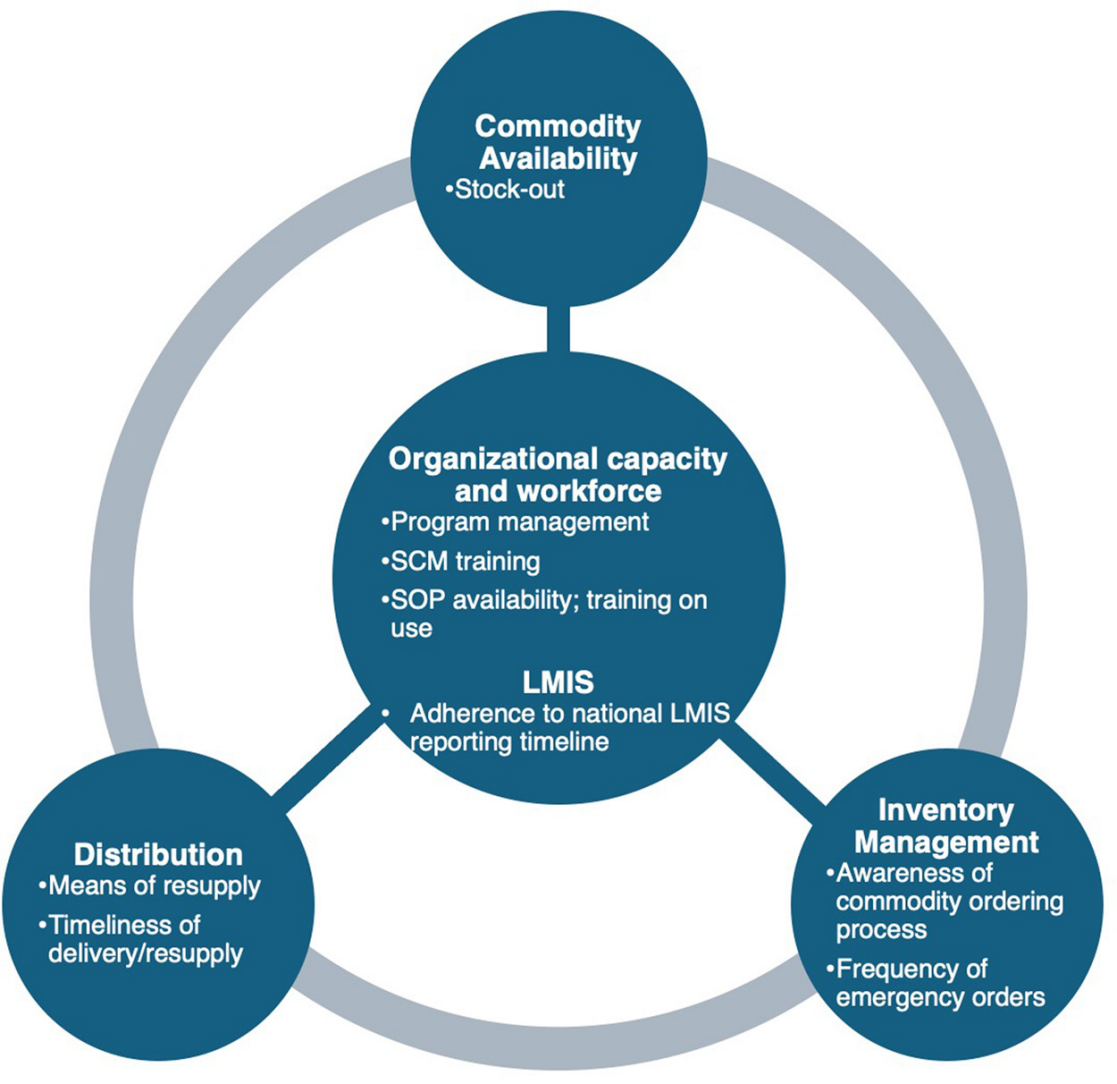
Conceptual Framework Showing Factors Influencing the Availability of Health Commodities Examined in the Study Abbreviations: LMIS, logistics management information system; SCM, supply chain management; SOP, standard operating procedure.

The study design was guided by the conceptual framework of factors that influence the availability of commodities (Figure 1). A skilled, highly motivated, and well-trained supply chain workforce is required to coordinate all actors and activities within the supply chain. This is reflected in this study by strong program management teams who train health care workers in SCM practices and who are then expected to use SCM standard operating procedures (SOPs) for commodity management. The application of the SOPs includes submitting good-quality LMIS reports in line with the national reporting timeline to inform regular and emergency resupply. The ordered products are then expected to be delivered on time through an established means of resupply to continuously meet patient needs (i.e., there should be no stock-out). Based on these factors, we assessed the following outcomes.

- Commodity availability:
  - Stock-out: The percentage of health facilities without usable stock of a given commodity on the assessment day.
- Organizational capacity and workforce:
  - SCM training: The percentage of health facilities with staff trained on the SCM of health commodities, including storage, inventory management, LMIS reporting, and distribution.
  - Availability and training on the use of SOPs for SCM: The percentage of health facilities with a copy of SOPs for SCM and the percentage of health facilities with staff trained on the use of the SOPs.
  - Program management and SCM challenges: The program management structure, including the situational assessment of LMCUs (i.e., operationalization of LMCUs, staff strength, and availability of functional budgets, among other considerations) and LMIS feedback mechanism (i.e., medium through which the LMCUs provided feedback to health facilities), procurement, and financing mechanisms, as well as logistics challenges across assessed facilities.
- Adherence to national LMIS reporting timeline: The percentage of health facilities submitting LMIS reports aligns with the national reporting timeline.
- Inventory management:
  - Awareness of commodity ordering process: The percentage of health facility staff who are aware of and can recollect the commodity ordering process for malaria commodities.
  - Frequency of emergency orders reported by health facilities.
- Distribution:
  - Means of resupply: The mode of resupply of commodities to health facilities.
  - Timeliness of delivery/resupply: The average time interval between when orders are placed and when commodities are delivered, as reported by health care workers. An average lead time of 2 weeks is considered timely.

### Data Analysis

Descriptive statistics in the form of counts and percentages were used to analyze quantitative data. After transcribing the qualitative data, participants’ responses were analyzed thematically. All analyses were conducted using Microsoft Excel.

### Ethical Approval

This study was approved by the designated authorities at the state ministries of health and state malaria elimination programs.

## RESULTS

### Commodity Availability

**FIGURE 2:**
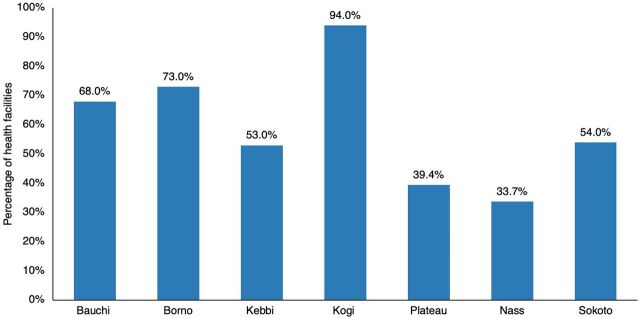
Percentage of Health Facilities Stocked Out of Malaria Rapid Diagnostic Tests, by State, Nigeria

**FIGURE 3:**
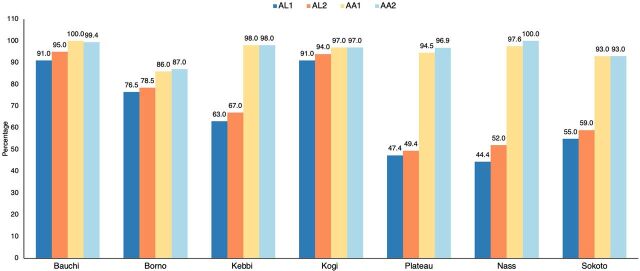
Percentage of Health Facilities Stocked Out of Artemisinin-Based Combination Therapies, by State, Nigeria Abbreviations: AL1, artemether 20 mg + lumefantrine 120 mg; AL2, artemether 20 mg + lumefantrine 120 mg; AA1, artesunate 25 mg + amodiaquine 67.5 mg; AA2, artesunate 50 mg + amodiaquine 135 mg.

In 5 of the 7 states, more than 50% of health facilities were stocked out of mRDTs, with the highest stock-out rate of 94% reported in Kogi State and the lowest stock-out rate of 33.7% in Nasarawa State (Figure 2). For all assessed ACTs, Bauchi and Kogi states had the highest stock-out rates of over 90% (Figure 3). Comparatively, AL combinations were more available than AA combinations across all 7 states.

### Organization Capacity and Workforce

The percentage of health workers that had received at least 1 form of SCM training ranged from 82% in Borno State to 93% in Bauchi and Sokoto states (Table 2). In all states except Borno State, the percentage of health facility staff trained in using SCM SOPs was higher than the percentage of health facilities with a copy of the SOPs (Table 2).

**TABLE 2.** Organizational SCM Capacity and Workforce

	**No. (%)**						
	**Bauchi**	**Borno**	**Kebbi**	**Kogi**	**Plateau**	**Nasarawa**	**Sokoto**
Health workers trained in SCM	312 (93)	164 (82)	266 (90)	158 (91)	241 (83)	221 (88)	279 (93)
SOPs available	215 (64)	166 (83)	211 (71)	114 (66)	175 (61)	164 (65)	239 (80)
Staff trained to use SOPs	292 (87)	144 (72)	275 (93)	142 (82)	236 (82)	218 (87)	274 (92)

Abbreviations: SCM, supply chain management; SOP, standard operating procedures.

Regarding the program management structure, all states and LGAs have LMCUs with 6–10 staff members (Table 3). The situational assessment of LMCUs revealed that only Borno and Bauchi states operated a drug revolving fund scheme. Respondents at the state level affirmed the dependence on donor funding for logistics activities and the use of electronic (national health logistics management information system) and paper-based LMIS.

**TABLE 3.** LMCU Situational Assessment, by State, Nigeria

	**Bauchi**	**Borno**	**Kebbi**	**Kogi**	**Plateau**	**Nasarawa**	**Sokoto**
Presence of functional LMCU							
State	Yes	Yes	Yes	Yes	Yes	Yes	Yes
LGA	Yes	Yes	Yes	Yes	Yes	Yes	Yes
No. LMCU staff	9	7	7	6	10	8	6
Public health commodities procurement							
Through SMOH, spearheaded by budget, planning, and public procurement	Yes	Yes	Yes	Yes	Yes	Yes	Yes
Drug revolving fund system	Yes	Yes	No	No	No	No	No
Logistics systems financing							
Mostly donor support	Yes	Yes	Yes	Yes	Yes	Yes	Yes
State budget	None	None	None	None	None	None	None
Push and pull distribution model, including redistribution between health facilities	Yes	Yes	Yes	Yes	Yes	Yes	Yes
NHLMIS platform and paper reporting	Yes	Yes	Yes	Yes	Yes	Yes	Yes

Abbreviations: LGA, local government area; LMCU, logistics management coordinative unit; NHLMIS, national health logistics management information system; SMOH, State Ministry of Health.

Respondents at the state level affirmed the dependence on donor funding for logistics activities.

Mechanisms for communicating feedback on LMIS reports included phone calls, (integrated) monitoring and supportive supervisory visits, coordination/review/data validation meetings, and the use of social media platforms, such as WhatsApp (Table 4). The top 2 logistics challenges were insecurity and finance.

**TABLE 4.** Feedback Mechanism on the Management of Malaria Commodities and Logistics Challenges

	**Bauchi**	**Borno**	**Kebbi**	**Kogi**	**Plateau**	**Nasarawa**	**Sokoto**
**State**							
Coordination/review/data validation meetings	No	No	No	Yes	Yes	No	No
IMSSVs	No	No	No	Yes	Yes	No	Yes
Phone call	No	Yes	Yes	No	No	Yes	Yes
WhatsApp platform	No	Yes	No	No	No	No	Yes
Written communication	No	No	No	No	No	No	Yes
Validated report	No	No	No	No	No	No	Yes
Stock/commodity tracker	Yes	No	No	No	No	No	No
Step down training	No	No	No	Yes	No	No	No
**LGA**							
Coordination/review/data validation meetings	Yes	Yes	No	Yes	Yes	No	No
IMSSVs	Yes	Yes	No	Yes	No	No	No
Phone call	Yes	No	No	No	Yes	No	No
WhatsApp platform	Yes	No	No	No	Yes	No	No
Step down training	No	No	No	Yes	No	No	No
**Logistics challenges**							
Labor disputes	No	No	No	No	Yes	Yes	No
Insecurity	No	Yes	Yes	Yes	Yes	No	Yes
Inadequate funding	Yes	No	Yes	Yes	No	No	Yes
Cultural festivals	No	No	No	Yes	No	No	No
Parallel operations	Yes	No	No	Yes	No	No	No
Territorial struggle	No	No	No	Yes	No	No	No

Abbreviations: IMSSV, integrated monitoring and supportive supervisory visits; LGA, local government area.

### Adherence to National Logistics Management Information System Reporting Timeline

Except for Kogi State, at least 76% of health facilities in all other states reported that they submitted LMIS reports every 2 months in line with the national reporting timeline (Table 5).

**TABLE 5.** LMIS Reporting Frequency Across Assessed Facilities, by State, Nigeria

	**No. (%)**						
	**Bauchi**	**Borno**	**Kebbi**	**Kogi**	**Plateau**	**Nasarawa**	**Sokoto**
Bimonthly	255 (76.0)	158 (79.0)	239 (81.0)	34 (20.0)	241 (83.4)	235 (93.3)	269 (90.0)
Quarterly	6 (2.0)	–	2 (1.0)	3 (2.0)	3(1.0)	2 (0.0)	–
Semiannually	–	–	1 (0.0)	–	–	–	1 (0.3)
Annually	15 (4.5)	–	–	1 (1.0)	–	–	2 (0.7)
Never^a^	59 (17.5)	–	54 (18.0)	134 (78.0)	45(15.6)	15(6.0)	27 (9.0)

Abbreviation: LMIS, logistics management information system.

^a^ Health facility has never reported or the respondent was not aware of the health facility ever submitting LMIS reports.

### Inventory Management

When asked about their awareness of the commodity ordering process, more than half of the respondents across all the states placed orders for malaria commodities using the national reporting tool for malaria commodities, the bimonthly facility stock report (Table 6). However, some health facility staff interviewed were unaware of the ordering process, while others reported other means.

**TABLE 6.** Commodity Inventory Management by State, Nigeria

	**%**						
	**Bauchi**	**Borno**	**Kebbi**	**Kogi**	**Plateau**	**Nasarawa**	**Sokoto**
**Awareness of commodity ordering process and medium**							
Through BFSR	57	73	69	69	72	87.4	86
Other means	29	19	15	15	15	6.3	4.0
Don’t know	14	8	16	16	13	6.3	10
**Emergency ordering frequency**							
Not placed in ≥ than 3 months	12.50	69	12	12	N/A	N/A	–
More than thrice in 3 months	–	3	–	–	N/A	N/A	–
Thrice in 3 months	–	7	–	–	N/A	N/A	–
Twice in 3 months		7	–	–	N/A	N/A	–
Once in 3 months		16	–	–	N/A	N/A	9

Abbreviations: BFSR, bimonthly facility stock report; N/A, not available.

Regarding the frequency of placing emergency orders, some health facilities reported placing emergency orders for malaria commodities across the state, with Sokoto State reporting the least (9%) (Table 6). The information on the percentage of health facilities that placed emergency orders was unavailable for Plateau and Nasarawa states.

### Distribution

**FIGURE 4:**
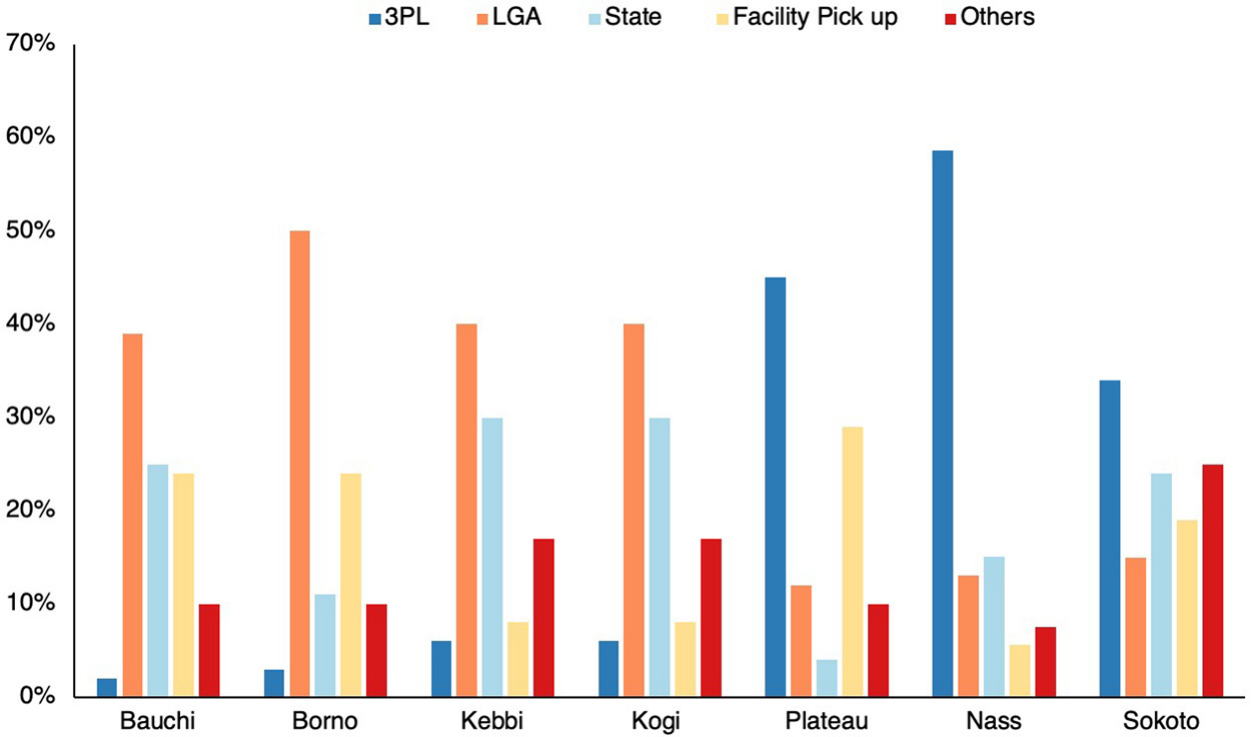
Means of Resupply of Health Facilities, by State, Nigeria Abbreviations: 3PL, third-party logistics; LGA, local government area; Nass, Nasarawa State

Regarding means of resupply of malaria commodities, higher percentages of health facilities in Plateau, Nasarawa, and Sokoto states received malaria commodities through a third-party logistics (3PL) service provider and appeared to have a lower proportion of health facilities with stock-out of malaria commodities at the time of visit (Table 7). In contrast, the LGA resupplied more health facilities in Bauchi, Borno, Kebbi, and Kogi states through direct delivery and, in some cases, pickup at the LGA by health facility representatives (Figure 4).

**TABLE 7.** Percentage of Health Facilities Resupplied Through 3PL and With Stock-Out of Malaria Commodities

	**Resupplied Through 3PL, %**	**With Stock-Out, %**				
		**RDT**	**AL1**	**AL2**	**AA1**	**AA2**
Bauchi	2.0	68.0	91.0	95.0	100.0	99.4
Borno	3.0	73.0	76.5	78.5	86.0	87.0
Kebbi	6.0	53.0	63.0	67.0	98.0	98.0
Kogi	6.0	94.0	91.0	94.0	97.0	97.0
Plateau	45.0	39.4	47.4	49.4	94.5	96.9
Nasarawa	58.7	33.7	44.4	52.0	97.6	100.0
Sokoto	34.0	54.0	55.0	59.0	93.0	93.0

Abbreviations: AL1, artemether 20 mg + lumefantrine 120 mg; AL2, artemether 20 mg + lumefantrine 120 mg; AA1, artesunate 25 mg + amodiaquine 67.5 mg; AA2, artesunate 50 mg + amodiaquine 135 mg.

In comparing the timeliness of commodity delivery, the percentage of health facilities that received malaria commodities within the recommended 2 weeks of placing an order was below average across most states except Sokoto and Bauchi states (Table 8).

**TABLE 8.** Timeliness and Lead Time for Resupply of Malaria Commodities

	**%**							
**Timely Delivery?**	**Lead Time**	**Bauchi**	**Borno**	**Kebbi**	**Kogi**	**Plateau**	**Nasarawa**	**Sokoto**
Yes	Less than 2 weeks	57	48	31	31	38	33	51
No	2 to 4 weeks	19	33	19	19	39	21	18
	4–8 weeks	11	11	32	32	18	39	25
	More than 2 months	13	9	19	19	5	8	6

## DISCUSSION

Our study showed that more than 50% of health facilities were stocked out of mRDTs on the day of the assessment in 5 northern Nigeria states. Similarly, stock-out rates for ACTs were over 50% for almost all assessed ACTs, with AL combinations being more available than AA combinations across all states. Earlier studies conducted in Nigeria have reported varying rates of malaria commodity stock-outs.^30^^,^^31^ For example, all public health facilities in Niger State were stocked out of mRDTs in the first quarter of 2012, with a stock-out rate of 76% or more for ACTs in the first 2 months of the same year.^30^ A 2014 assessment across 252 health facilities in Nigeria yielded a stock-out rate of 28% for AL1,^32^ which is considerably lower than our finding, suggesting that the malaria commodity availability situation has worsened since then. The disproportionate availability of AL when compared with AA could negatively impact the implementation of multiple first-line therapies, a desired strategy to increase the therapeutic life of most ACTs.^33^ The poor availability of mRDTs and ACTs is detrimental to managing uncomplicated malaria among children aged younger than 5 years, especially among individuals who predominantly access treatment through public health facilities.^33^^–^^35^ This poor availability may have accounted for nonadherence to the malaria treatment guidelines, which have been reported in several studies.^32^^,^^34^^,^^35^ The stock-out of mRDTs was recently identified as a major reason for the poor implementation of the test and treat policy^36^ in sub-Saharan Africa.^37^ Due to the low availability of malaria commodities in the public sector, some individuals may have sought treatment from the private sector (e.g., private hospitals and clinics, pharmacies, and proprietary and patent medicine vendors), which accounts for 70% of health care services provision in Nigeria.^38^ This may put pressure on household income, as individuals typically pay out of pocket for private sector services.^39^

The poor availability of mRDTs and ACTs may have accounted for nonadherence to the malaria treatment guidelines shown in several studies.

Overall, the poor availability of malaria commodities raises the need for more investment in strengthening the public health supply chain, especially at the subnational level, to reduce malaria-related morbidity and mortality. Our assessment yielded insights into some factors that can impact malaria commodity availability. Starting with coordination and the supply chain workforce, all states and LGAs had LMCUs that provided feedback to health facilities through in-person and online media. Established with support from donors, this coordination structure can enhance the coordination of SCM activities.^11^^,^^40^^,^^41^ Although establishing LMCUs at the subnational level is commendable, the top 2 logistics challenges identified were insecurity and inadequate funding. Other studies have also identified poor funding as a major SCM challenge in Nigeria.^22^^,^^42^

The lack of funding and, in some cases, non-release or delayed release of funding for these units could, however, limit their ability to deliver on their mandates, one of which is monitoring and supportive visits, which can improve the SCM practices at the health facilities. Their use of virtual mediums (e.g., phone calls and WhatsApp) to provide LMIS feedback is commendable, especially where movement is restricted (e.g., during the COVID-19 pandemic) or funding is limited for in-person engagement.^43^ However, these alternative modes should be viewed as complementary, as in-person supportive supervisory visits may still be needed to mentor health workers on completing quality LMIS reports and other SCM best practices. Other countries may want to consider the establishment of LMCUs as part of the broader initiatives to strengthen SCM.

In this study, more than 80% of staff at the health facility had received at least 1 form of SCM training and had been trained on using SCM SOPs, except in Bauchi State. However, only 2 states (Borno and Sokoto states) had 80% or more of health facilities with SCM SOPs. Trained health care workers are needed to effectively and efficiently manage health commodities, and the use of SOPs can facilitate this. For example, using SOPs can support accurate ordering that could prevent stock-outs by providing the needed instructions, especially as the malaria commodity system in Nigeria uses a pull logistics system, where the responsibility of correctly determining order quantities lies with the health facilities.^44^ Therefore, it is crucial for SOPs to be available across all health facilities. Stakeholders should ensure that capacity gaps, as identified in this study, are addressed through training and mentorship while making SOPs available across all health facilities to guide health workers. It is equally important to note that the current LMIS form used by the malaria program does not take seasonality into account, and this may result in an underestimation of commodity requirements and, in turn, stock-outs.^45^ Also, labor disputes are one of the SCM challenges that can and have historically disrupted service delivery in Nigeria, including access to malaria health care services, underscoring the need to establish measures to avert or promptly resolve labor disputes to ensure that health workers stay motivated.^46^^–^^48^

Stakeholders should ensure that workforce capacity gaps are addressed through training and mentorship and make SOPs available across all health facilities to guide health workers.

More than half of the respondents placed orders using the national reporting tool, with LMIS reports submitted every 2 months across more than 76% of health facilities. In general, states (Plateau, Nasarawa, and Sokoto states) with a higher percentage of health workers who were aware of the commodity ordering process, adhered to the national LMIS reporting timeline, and had a predominantly 3PL service provider-driven commodity delivery system tended to have higher availability of mRDTs and AL combinations. This is not surprising, given that placing orders following the approved national system can facilitate timely resupply, especially for health facilities benefiting from the free malaria commodities program. Similarly, the use of 3PL service providers has been shown to improve commodity supply because it allows the government to harness the expertise and efficiencies available within the private sector. The study findings suggest that using 3PL service providers for distribution should be encouraged where it is deemed sustainable, given the funding and oversight capacity requirements.^49^ This benefit is further enhanced for the malaria program by bypassing the intermediary stores (i.e., commodities are delivered directly to health facilities from the central or zonal stores).^44^^,^^50^ Despite these advantages, the percentage of health facilities that received malaria commodities within the recommended 2 weeks of placing an order was below average across most states, except Sokoto and Bauchi states.

### Strength and Limitations

The main strength of this study is the coverage of a considerable number of health facilities across all the northern geopolitical zones of Nigeria and the incorporation of subnational stakeholders’ opinions compared to other studies conducted in the country. However, the findings from our study should be interpreted considering the following limitations. First, the availability of commodities was observed on the assessment day and may not necessarily represent the average product availability over time. However, measuring availability on the survey’s day remains the most logistically feasible and justified, given that these commodities are expected to be always available. Second, the lack of commodities does not equate to a complete lack of access, as caregivers can still purchase these commodities from the private sector (pharmacies and patent medicine stores). Also, qualitative responses from participants were not triangulated with LMIS responses. Lastly, we did not examine national-level challenges and other distal supply chain functions, such as poor quantification, inadequate funding for procurement, or bureaucratic bottlenecks within the public health institutions that could also affect product availability.^51^^–^^53^

## CONCLUSION

The availability of lifesaving malaria commodities across the health facilities visited was suboptimal. Several SCM challenges that may be responsible for poor availability were also identified. Once causality is established, effective interventions to address SCM gaps need to be implemented to contribute to reducing malaria morbidity and mortality in Nigeria among children aged younger than 5 years.

## Supplementary Material

GHSP-D-22-00547-supplement.pdf

## Notes

**Peer Reviewed**

**First published online:** June 13, 2024
