# Supplementary material for: Examining Public Sector Availability and Supply Chain Management Practices for Malaria Commodities: Findings From Northern Nigeria
Source: Glob Health Sci Pract. 2024 Jun 27;12(3):e2200547. doi: 10.9745/GHSP-D-22-00547 (PMC11216708; doi:10.9745/GHSP-D-22-00547)
Supplement: GHSP-D-22-00547-supplement.pdf [file GHSP-D-22-00547-supplement.pdf]

Supplement to: Rotimi K, Itiola AJ, Fagbemi BA, et al. Examining public sector availability and supply chain management practices for malaria commodities: findings from northern Nigeria. *Glob Health Sci Pract*. 2024;12(3):e2200547. https://doi.org/10.9745/GHSP-D-22-00547

TOOLS FOR THE STUDY

Stock Sheet for Stock Count

S/N	Commodities	Physical Count	Date of Expiry
1	AL1		
2	AL2		
3	AA1		
4	AA2		
5	RDT		

Supplement to: Rotimi K, Itiola AJ, Fagbemi BA, et al. Examining public sector availability and supply chain management practices for malaria commodities: findings from northern Nigeria. *Glob Health Sci Pract*. 2024;12(3):e2200547. https://doi.org/10.9745/GHSP-D-22-00547

QUESTIONNAIRES

**Commodity Management Audit Form - 2021- State Level Questions**
Q1. State.
Q2. GPS
Q1. State.
**SNAP LSAT Questions**
Does the state/LGA level have a logistics management unit?
Is the logistics management unit fully responsible for the following activities? (If not, note the departments or positions responsible for each logistics task.):
- managing and using the logistics management information system?
- forecasting quantities needed?
- procurement?
- inventory management, storage, and distribution?
- product selection?
- staffing of logistics positions?
- budgeting for the logistics system?
Supervision and logistic staff development?
Are there documented guidelines for logistics activities?
- managing and using the logistics management information system?
- forecasting quantities needed?
- procurement?
- inventory management, storage, and distribution?
- product selection?
- staffing of logistics positions?
- budgeting for the logistics system?
Supervision and logistic staff development?

Supplement to: Rotimi K, Itiola AJ, Fagbemi BA, et al. Examining public sector availability and supply chain management practices for malaria commodities: findings from northern Nigeria. *Glob Health Sci Pract*. 2024;12(3):e2200547. https://doi.org/10.9745/GHSP-D-22-00547

How many personnel positions have key logistics tasks?
How many of the positions with key logistics tasks are currently filled? If they are not filled, why not?
Provide or map an organogram that includes key stakeholders' relationships, including government units, donors, other cooperating agencies, and other supply chains (responsibilities for logistics activities).
What issues outside the supply chain impact the functioning of the supply chain? (Note: Include major political, cultural, or economic factors, such as political events, labour disputes.)
Is there a state-level policy on the malaria program being assessed?
Does the state policy address commodity security? Please explain.
Does the policy influence the annual planning process? Please explain.
Is there a logistics management information system?
Is logistics information collected through another information system (e.g., HMIS)? Describe briefly.
Does the information system (LMIS, HMIS, other) include?
- stock keeping records (e.g., inventory control cards, bin cards, stock registers) at all levels?
- requisition and issue records (e.g., bills of lading, shipping records, requisition/issue
vouchers) at all levels?
Dispensed-to-user records at service delivery points?
Describe the flow of information from the health facility to the state level. Please include information about forms used, frequency of reporting, who's responsible, and where data is aggregated. Attach a diagram.
Do LMIS or other information system reports received at the state level provide information on stock status at the health facility level (i.e., do state-level staff have accurate routine information on which facilities are stocked out, understocked, adequately stocked, or overstocked)? Please explain
How often are reports sent to each higher level of the system? Map the report flow.
Are information system records reconciled against physical inventories at each level?
If yes, how are information system records reconciled against physical inventories at each level?
Is the information system automated at the state level?
If yes, briefly describe the process that is automated.
Is the information system used to monitor and evaluate the program's performance?

Supplement to: Rotimi K, Itiola AJ, Fagbemi BA, et al. Examining public sector availability and supply chain management practices for malaria commodities: findings from northern Nigeria. *Glob Health Sci Pract*. 2024;12(3):e2200547. https://doi.org/10.9745/GHSP-D-22-00547

What indicators related to logistics and product availability does the information system track (e.g., stockout rate, percentage of reporting, rational prescribing practices.)?
What feedback mechanisms are in place to channel logistics information back to lower levels?
How does the state manage the stockout of malaria commodities?
Specify what type of inventory control system is used (e.g., push, pull.) and describe the system. Draw/attach a diagram showing the relationships between the various levels.
Are there written provisions for the redistribution of over-stocked supplies?
How are products delivered between each level of the system (including frequency and means of transportation)? Specify between which levels. How are routes determined?
Are vehicles regularly available for supervision?
How often is supervision conducted at the service delivery points?
Is external assistance (from other NGOs, donors, or partners) used to complete management and supervision activities?
Who finances the state logistics program's annual budget?
Is there a donor coordination committee? How often do they meet?
Any other comment

Supplement to: Rotimi K, Itiola AJ, Fagbemi BA, et al. Examining public sector availability and supply chain management practices for malaria commodities: findings from northern Nigeria. *Glob Health Sci Pract*. 2024;12(3):e2200547. https://doi.org/10.9745/GHSP-D-22-00547

**LGA Level**
Q1. State.
Q2. LGA.
03. GPS
**_Audit Questions**
**SNAP LSAT Questions**
Does the LGA level have a logistics management unit?
Is the logistics management unit fully responsible for the following activities? (If not, note the departments or positions responsible for each logistics task.):
- managing and using the logistics management information system?
- forecasting quantities needed?
- procurement?
- inventory management, storage, and distribution?
- product selection?
- staffing of logistics positions?
- budgeting for the logistics system?
- supervision and logistic staff development?
Are there documented guidelines for logistics activities?
- managing and using the logistics management information system?
- forecasting quantities needed?
- procurement?
- inventory management, storage, and distribution?
- product selection?
- staffing of logistics positions?
- budgeting for the logistics system?
- Supervision and logistic staff development?

Supplement to: Rotimi K, Itiola AJ, Fagbemi BA, et al. Examining public sector availability and supply chain management practices for malaria commodities: findings from northern Nigeria. *Glob Health Sci Pract*. 2024;12(3):e2200547. https://doi.org/10.9745/GHSP-D-22-00547

What issues outside the supply chain impact the functioning of the supply chain in the LGA? (Note: Include major political, cultural, or economic factors, such as political events, labour disputes).
Is there a logistics management information system?
Is logistics information collected through another information system (e.g., HMIS)? Describe briefly.
Do LMIS or other information system reports received at this level provide information on stock status at the health facility level (i.e., do LGA-level staff have accurate routine information on which facilities are stocked out, understocked, adequately stocked, or overstocked)? Please explain
How often are reports sent to each higher level of the system? Explain the report flow.
How do managers monitor reporting rates and follow-up to obtain missing logistics reports?
Is the information system automated at this level?
If yes, briefly describe the process that is automated.
What indicators related to logistics and product availability does the information system track (e.g., stockout rate, percentage of reporting, rational prescribing practices, etc.)?
What feedback mechanisms are in place to channel logistics information back to lower levels?
Are there written provisions for the redistribution of over-stocked supplies within the LGA?
Are vehicles regularly available for supervision?
How often is supervision conducted at the service delivery points?
Any other comment
**How many Contact Persons**
**List all the contact persons by adding a group.**
**contact person**
Member ID number
Name
Phone Number
Designation

Supplement to: Rotimi K, Itiola AJ, Fagbemi BA, et al. Examining public sector availability and supply chain management practices for malaria commodities: findings from northern Nigeria. *Glob Health Sci Pract*. 2024;12(3):e2200547. https://doi.org/10.9745/GHSP-D-22-00547

**Health Facility Level**
**SMA_Audit Questions**
Q1. State.
Q2. LGA.
Q3. Ward
Q4. Facility
Q5. GPS
Are ICC available for AL 1 in the store?
Are ICC available for AL2 in the store?
Are ICC available for AA1 in the store?
Are ICC available for AA2 in the store?
Are ICC available for RDTS in the store?
**SNAP LIAT Questions**
Who is the main person responsible for managing medical supplies at this facility?
- Nurse
- Pharmacy technician
- Pharmacy assistant
- Pharmacist
- Medical assistant
- CHO
- SCHEW/CHEW
- JCHEW
- Others (specify)
Have you or any of the other HF staff had formal logistics training? (i.e., ordering, receiving, inventory management)
If yes, state the training type and year.
- Logistics Management of malaria commodities 2020 or 2021

Supplement to: Rotimi K, Itiola AJ, Fagbemi BA, et al. Examining public sector availability and supply chain management practices for malaria commodities: findings from northern Nigeria. *Glob Health Sci Pract*. 2024;12(3):e2200547. https://doi.org/10.9745/GHSP-D-22-00547

- Logistics Management of malaria commodities 2019
- Logistics Management of SPAQ commodities 2019 or 2021
- Others
Are there any printed logistics guidelines available for use in the HF? (*Ask to see a copy & please give details (e.g., title, date)*
Have you or any other HF staff received training on the use of any guidelines and SOP for malaria commodity logistics?
If No, state how staff learned to use the SOPs.
Are Daily Consumption Register available - *ask to see a copy*
Are Bimonthly Facility Stock report form available- *ask to see a copy*
Are Return &Transfer Form available - *ask to see a copy*
Do you submit the BFSR report with logistics data? (*Check the BFSR and record yes if the form is complete*
- How often do you submit these reports?
- Bimonthly
- Quarterly
- Semi-annually
- Annually
- Never
How did you learn to complete the forms/records used at this facility?
- Never learned
- During a logistics workshop
- On the job training
- On the job (self-learning)
- Other (Specify):
How many emergency orders for antimalarials have you placed in the last three months?
- None
- One

Supplement to: Rotimi K, Itiola AJ, Fagbemi BA, et al. Examining public sector availability and supply chain management practices for malaria commodities: findings from northern Nigeria. *Glob Health Sci Pract*. 2024;12(3):e2200547. https://doi.org/10.9745/GHSP-D-22-00547

- Two
- Three
- >Three
How are the facility's re-supply quantities determined?
- through Bimonthly SFR report
- Don't know
- Other means
Who is responsible for transporting malaria products to your facility?
- 3PL
- LGA
- State
- This facility collects
- Other (*Specify*):
Approximately how long does it take between reporting/ordering and receiving products?
- < 2 weeks
- Two weeks – 1 month
- 1 - 2 months
- > 2 months
When did you receive your most recent general supervision visit? Check visitor's book if necessary.
- Never received
- Within the last month
- Within the last three months
- Within the last six months
- > 6 months ago,
- Other (Specify)
Is there a Health Committee at this facility?

Supplement to: Rotimi K, Itiola AJ, Fagbemi BA, et al. Examining public sector availability and supply chain management practices for malaria commodities: findings from northern Nigeria. *Glob Health Sci Pract*. 2024;12(3):e2200547. https://doi.org/10.9745/GHSP-D-22-00547

When was the last Health Committee meeting?
What are the biggest challenges to you in managing the medicines supply system?
What would you like to be done to address these?
Any other comments?
**SPAQ Storage Assessment**
Is the store well-located (easily accessible for receipts & issues)
Are SPAQ and ACT stored separately from insecticides and chemicals?
Is the storeroom free of rodents or insects in the storage area?
Is storage area is secured with a lock and key
Access is limited to authorized personnel in the stores
Are SPAQ and ACT protected from direct sunlight?
Are SPAQ and ACT stored at room temperature?
Is there a fire extinguisher or sand bucket available at the store?
Are stocks stored off the floor on pallets/shelves/ tables?
Are stocks well-positioned with labels and expiry dates and manufacturing dates clearly visible?
Are stocks systematically organized to facilitate easy location, FEFO, and good management?
Are damaged and deteriorated commodities kept with usable stock?
Are expired items removed and kept well separated from usable items pending disposal?
Are Flammable items stored correctly (i.e. separate, cool place)
Any comment
**Contact person**
Member ID number
Name
Phone Number
Designation
